# The Separation, Purification, Structure Identification, and Antioxidant Activity of *Elaeagnus umbellata* Polysaccharides

**DOI:** 10.3390/molecules28186468

**Published:** 2023-09-06

**Authors:** Jinhua Zhang, Xin Xu, Xinyi Liu, Min Chen, Baoqing Bai, Yukun Yang, Tao Bo, Sanhong Fan

**Affiliations:** 1 School of Life Science, Shanxi University, Taiyuan 030006, China; ever840605@sxu.edu.cn (J.Z.); xu1784407579@163.com (X.X.); qq85242354@126.com (X.L.); baoqingbai@sxu.edu.cn (B.B.); yangyukun@sxu.edu.cn (Y.Y.); 2 Shanxi Key Laboratory for Research and Development of Regional Plants, Shanxi University, Taiyuan 030006, China; 3 Shanxi Food Research Institute, Co., Ltd., Taiyuan 030024, China; chenmin768@126.com; 4 Institute of Biotechnology, Shanxi University, Taiyuan 030006, China

**Keywords:** *Elaeagnus umbellata*, polysaccharides, antioxidant activity, functional food

## Abstract

In order to investigate the antioxidant activity of *Elaeagnus umbellata* polysaccharides, the physicochemical characteristics of purified *Elaeagnus umbellata* polysaccharides (EUP, consisting of two fractions, EUP1 and EUP2) were investigated using UV spectrophotometry, high-performance liquid chromatography (HPLC), high-performance gel permeation chromatography (HPGPC), and Fourier transform infrared spectroscopy (FT-IR). This revealed that EUP1 and EUP2 were acidic polysaccharides with an average molecular weight (MW) of 63 and 38 kDa, respectively. EUP1 mainly consisted of L-rhamnose and D-galactose in a molar ratio of 2.05:1, and EUP2 consisted of D-mannose, L-rhamnose, D-galactose, and D-arabinose in a molar ratio of 2.06:1:2.78:1. Furthermore, EUP exhibited considerable antioxidant potential for scavenging hydroxyl, superoxide anion, DPPH, and ABTS radicals. Therefore, EUP can be developed as a potential antioxidant for the functional food or pharmaceutical field.

## 1. Introduction

*Elaeagnus umbellata* Thunb., known as the autumn olive, belongs to the Elaeagnaceae family [1], which consists of approximately 90 species and is found at altitudes between 1200 and 2100 m, mostly in regions of Asia [2,3]. Several species of Elaeagnus, such as *E. umbellata*, *E. pungens*, *E. angustifolia*, and *E. multiflora*, have been used as medicinal plants [2,3]. *E. umbellata* is a large, spreading, spiny-branched shrub, 3.5 to 5.5 m high and 3.5 to 5.5 m wide. Its foliage is light green on top and silvery green underneath. Its fleshy fruit is of a sub-globose to broadly ellipsoid shape, 6 to 8 mm long. Individual fruits are 1.25 to 1.5 cm in size with a spotted light-green color in mid-summer, turning red in the autumn [4]. A mature plant can produce between 0.9 and 3.4 kg of fruit each year, with the number of seeds ranging between 20,000 and 54,000 [5].

Polysaccharides are long-chain polymers formed by more than 20 monosaccharide molecules linked together by glycosidic bonds. Naturally extracted polysaccharides have a wide range of sources and mild properties. They are a very important class of macromolecular substances in higher plants, animals, and microbial cells [6]. Food-derived polysaccharides refer to natural polysaccharides with complex structures extracted from edible resources. They have multiple functional activities, and their sources include foods in people’s daily lives, such as fruits, vegetables, grains, aquatic products and other meat products. 

The research on *E. umbellata* has mainly focused on its ecological learning, nutritional composition analysis (in terms of tissue culture), separation and determination of medicinal components; there are few research reports on *E. umbellata* tree polysaccharides. Plant polysaccharides have functions such as antioxidation, anti-aging, anti-tumor, and immune regulation. These functions are mainly related to the antioxidation effect of polysaccharides. By comparing the in vitro antioxidant effects of polysaccharides from different parts of the *E. umbellata* tree, the results showed that polysaccharides from different parts of the *E. umbellata* tree have scavenging or inhibitory effects on hydroxyl radicals and superoxide anion radicals and have a good dose–effect relationship. Through comparison, it has been found that the antioxidant activity of polysaccharides in the fruit is stronger than that of the other two parts—the leaves and the branches. It is inferred that the composition of the polysaccharides in different parts of the *E. umbellata* tree is different, and the two kinds of free radicals react. The polysaccharide composition of the different parts of the system may not be completely the same. For this reason, further in-depth study of the polysaccharide composition of each part is needed [7]. 

In this study, its fruit was selected as the preferred part for screening natural antioxidants. *E. umbellata* is well known because of the lycopene content of its berries, which provides antioxidant and anti-inflammatory activities [8]. However, the physiological activity of its polysaccharides has not yet been explored because their contents are relatively less than that of lycopene. Therefore, the polysaccharide from *E. umbellata* was isolated and purified using various methods to analyze the structure and molecular morphology of the polysaccharide and to study its antioxidant activity. This study aims to investigate the structural characteristics and antioxidant activity of *E. umbellata* polysaccharides to provide a theoretical basis for their development and utilization. 

## 2. Results 

### 2.1. The Separation and Purification of CEP

#### 2.1.1. Purification of CEP

**Figure 1:**
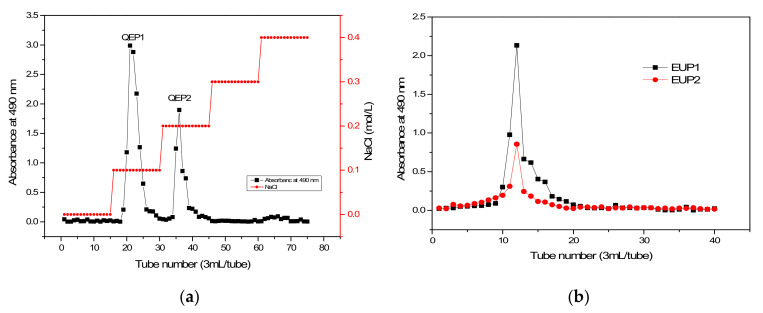
Elution curves of crude polysaccharide fractions on the DEAE Cellulose-52 column ((**a**): QEP1 and QEP2) and the Sephadex G-100 gel column ((**b**): EUP1 and EUP2).

The purpose of separation and purification is to remove monosaccharides, oligosaccharides, pigments, proteins, minerals, and toxic components. In this study, classic deproteinization methods and the neutral protease + Sevag method were evaluated for the crude polysaccharides (CEPs) processing. A DEAE-52 column has the advantages of high chemical stability, high loading capacity and long service time, but it needs to be regenerated after use to remove impurities such as pigment. A Sephadex-G100 column has the characteristics of small non-specific adsorption, high recovery rate and bacteriostasis [9]. The CEPs extracted from *Elaeagnus umbellata* were purified by DEAE-52 anion-exchange chromatography, resulting in two elution peaks, known as QEP1 and QEP2 (QEP1 and QEP2 in Figure 1a). The two fractions were obtained using Tris-HCl, 0 M NaCl, 0.1 M NaCl, 0.2 M NaCl, 0.3 M NaCl, and 0.4 M NaCl as the eluent. The yields of QEP1 and QEP2 were 53.38% and 28.13%, respectively, amounting to an overall recovery of 81.51% of the crude polysaccharides applied to the column, suggesting that the elution was effective.

The purified polysaccharide fractions (QEP1 and QEP2) were further purified using Sephadex G-100 chromatography. Figure 1b shows that only one peak was obtained from each fraction. The yields of EUP1 and EUP2 were 43.68% and 20.85%, respectively. The two fractions were collected for further structural and activity analyses.

#### 2.1.2. Analysis of the UV and FT-IR Spectra

**Figure 2:**
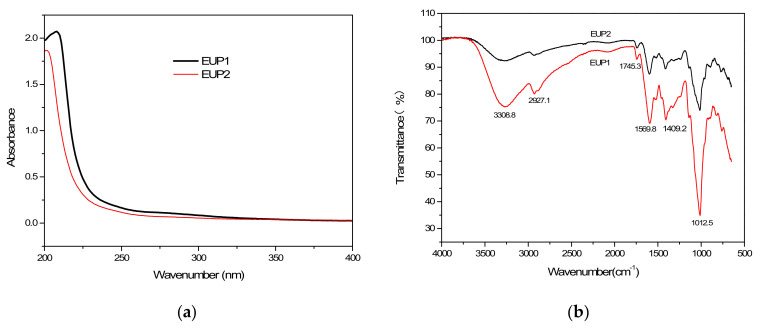
Characterization of the polysaccharide fractions: (**a**) UV absorption spectra of EUP1 and EUP2; (**b**) FT−IR spectra of EUP1 and EUP2.

The biological activities of polysaccharides are closely related to their structures. Therefore, it is of great significance to elucidate the structural characteristics and biological activities of polysaccharides to explore the structure–activity relationship between polysaccharide structure and biological activities. Figure 2a shows that the UV spectra exhibited no absorption peaks at 260 and 280 nm for the major fractions (EUP1 and EUP2), indicating the absence of nucleic acids and proteins. The FT-IR spectroscopy revealed the major functional groups of the polysaccharides and the similar spectral characteristics of the two polysaccharide fractions (Figure 2b). Both polysaccharides extracted from *Elaeagnus umbellata* exhibited the typical absorption bands at 3308.8 cm^−1^ from O–H stretching in the sugar residues [10,11], and a weak absorption band at 2927.1 cm^−1^ for C–H stretching in the CH_2_ groups [12].

Generally, a band at 1740 cm^−1^, characteristic of uronic acid, is used to determine the degree of polysaccharide esterification [13]. The weak absorption band peak at 1745.3 cm^−1^ indicated the presence of carboxylic groups in EUP1 and EUP2 [14]. In particular, a specific absorption peak at 1569.8 cm^−1^ from N–H bending in the polysaccharide in both polysaccharide fractions suggested that hydroxyl groups had been substituted, indicating a decrease in its polarity [15,16]. The stretching peaks at 1409.2 cm^−1^ from O–H deformation could be attributed to symmetric COO–, indicating the presence of uronic acid in both polysaccharides [17]. Strong peaks between 1200 and 1000 cm^−1^ indicated the presence of a C–O–H side group and C–O–C glycosidic bond vibrations [18], with a strong peak at 1012.5 cm^−1^ indicating an α-1,6 linkage [19]. 

#### 2.1.3. Analysis of the Molecular Weights of the Purified Polysaccharides 

**Figure 3:**
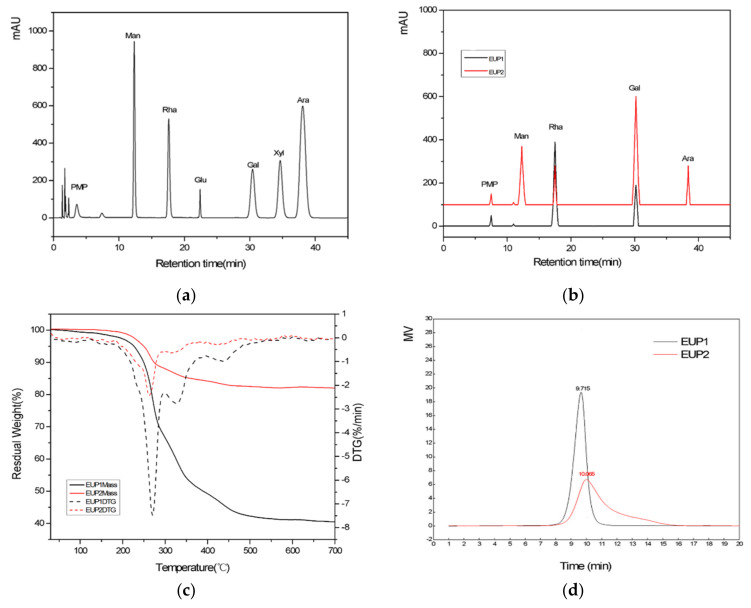
(**a**) HPGPC graph of EUP1 and EUP2; (**b**) HPLC chromatogram of the monosaccharide standards; (**c**) HPLC chromatogram profiles of the monosaccharide composition of EUP1 and EUP2; and (**d**) TGA (Thermal gravimetric Analysis) and DTA (Differential Thermal Analysis) curves of EUP1 and EUP2.

The MW of EUP1 and EUP2 were determined to use the HPGPC method. A standard curve was plotted using the dextran standards (10, 40, 50, 70, 500, and 2000 kDa). The MW of EUP1 and EUP2 were determined to use the standard curve [20]. The chemical structure of a polysaccharide also depends on its degree of polymerization as well as its functional groups; so, these were characterized for both polysaccharides by determining their MW to verify their structural characteristics and extraction behaviors. The single chromatographic peaks proved that the two polysaccharides were relatively homogeneous polysaccharides [21]. Elution times of 9.715 and 10.065 min were observed for EUP1 and EUP2, respectively (Figure 3a), leading to MW estimates of 63 and 38 kDa, respectively, from the calibration curve. This significant difference in MW between the two polysaccharide fractions indicated that the chemical structure of EUP1 was more highly polymerized than that of EUP2. 

The MW of the two polysaccharides was less than of those obtained by traditional hot-water extraction (600–23,600 kDa) [22], possibly because homogenate extraction or enzymatic hydrolysis reduces the MW of polysaccharides.

#### 2.1.4. Monosaccharide Component Analysis

The composition and content changes of the monosaccharides will directly affect the biological activity of the polysaccharides. By conducting monosaccharide composition analysis, the results showed that EUP1 consisted of two monosaccharides, L-rhamnose and D-galactose, and EUP2 consisted of four monosaccharides, D-mannose, L-rhamnose, D-galactose, and D-arabinose, thus demonstrating the different chemical compositions of the two polysaccharides. There were also differences in the molar ratio composition and mass percentages of the monosaccharides. For example, the contents of D-arabinose and D-galactose in both polysaccharides were clearly different, leading to differences in the molar ratio and polarity. The molar ratios of L-rhamnose and D-galactose were 2.05:1 for the polysaccharide in EUP1, and those of D-mannose, L-rhamnose, D-galactose, and D-arabinose were 2.06:1:2.78:1 for the polysaccharide in EUP2. In particular, the introduction of more aminos from the galactose would increase the polarity of the polysaccharide of EUP2.

#### 2.1.5. Scanning Electron Microscopy (SEM)

**Figure 4:**
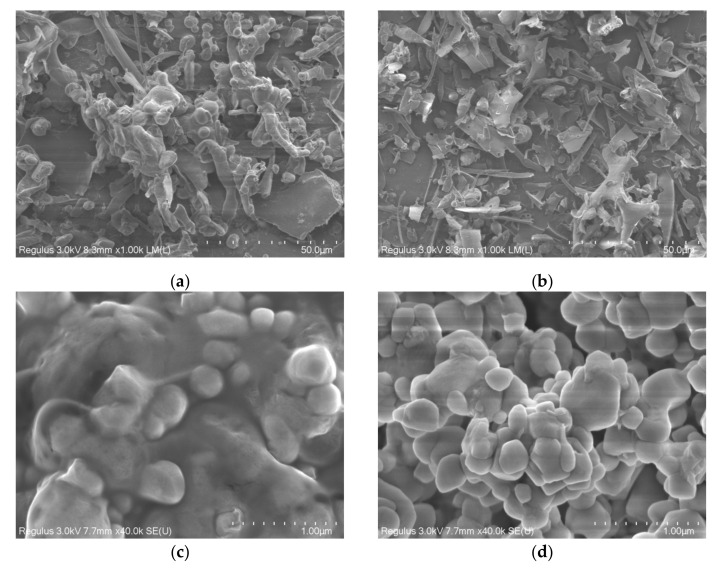
SEM micrographs of EUP1 and EUP2. (**a**) Morphology of EUP1 (×1000); (**b**) morphology of EUP2 (×1000); (**c**) morphology of EUP1 (×40,000); and (**d**) morphology of EUP2 (×40,000).

The three-dimensional structures of polysaccharides are typically more complex than those of nucleotides and proteins. To provide direct evidence for the conformations of the chains in EUP1 and EUP2, SEM was used to examine the morphologies of the polysaccharides [23]. The purified polysaccharides were mainly composed of irregular and fragmented structures of different sizes. EUP1 exhibited an irregular strip structure interspersed with many relatively large spherical particles (Figure 4a,c), whereas EUP2 exhibited a rough flaky structure interspersed with many small spherical particles (Figure 4b,d). In comparison, the EUP1 polysaccharides exhibited a strong interaction between components with their closely bound structure, whereas the EUP2 polysaccharides were incompletely bound together, with small gaps in the structure. This indicated that there was repulsion between the polysaccharide molecules, and that any intermolecular attraction was small.

#### 2.1.6. Thermal Gravimetric Analysis (TGA)

The weight loss patterns of the EUP1 and EUP2 complex polymers as a function of temperature are shown in Figure 3d. The TGA curves exhibited patterns of degradation consisting of three steps. The first step of mass loss occurred between 65 and 150 °C, possibly related to the evaporation of bound water. Small variations in the mass of EUP1 were observed between 65 and 150 °C because of water evaporation, but EUP2 exhibited almost no variations in mass. The second and major step of mass loss occurred between 240 °C and 456 °C and corresponded to the depolymerization of the polysaccharide structure. EUP1 and EUP2 started to decompose at 249.1 °C and 241.0 °C, respectively, when their weight decreased sharply by 56.46% and 16.98%, respectively, with a DTG (derivative thermogravimetry) minimum at degradation temperatures of 270.2 °C and 262.6 °C, respectively. The third step of mass loss between 456 °C and 600 °C was related to the oxidation of organic matter, where EUP1 exhibited small variations in mass but EUP2 did not. The thermogram indicates that the polysaccharides were chemically stable (Figure 3d). The thermal stability of polysaccharides depends on variations in their structural and functional groups. The DTG thermograms showed that the maximum rate of weight loss occurred at 270.2 °C for EUP1 and at 262.6 °C for EUP2. The high MW of EUP2 may also be considered to be responsible for these differences in the thermal decomposition temperature. These results suggested that EUP2 had a more stable structure than EUP1 [24].

### 2.2. Antioxidant Activities of EUP1, EUP2, and CEP

#### 2.2.1. DPPH Free Radical Scavenging Ability

**Figure 5:**
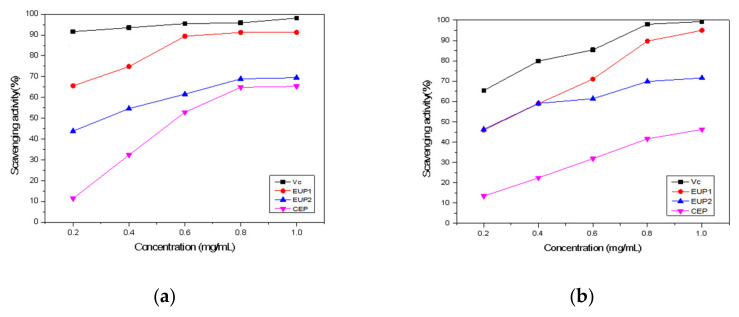

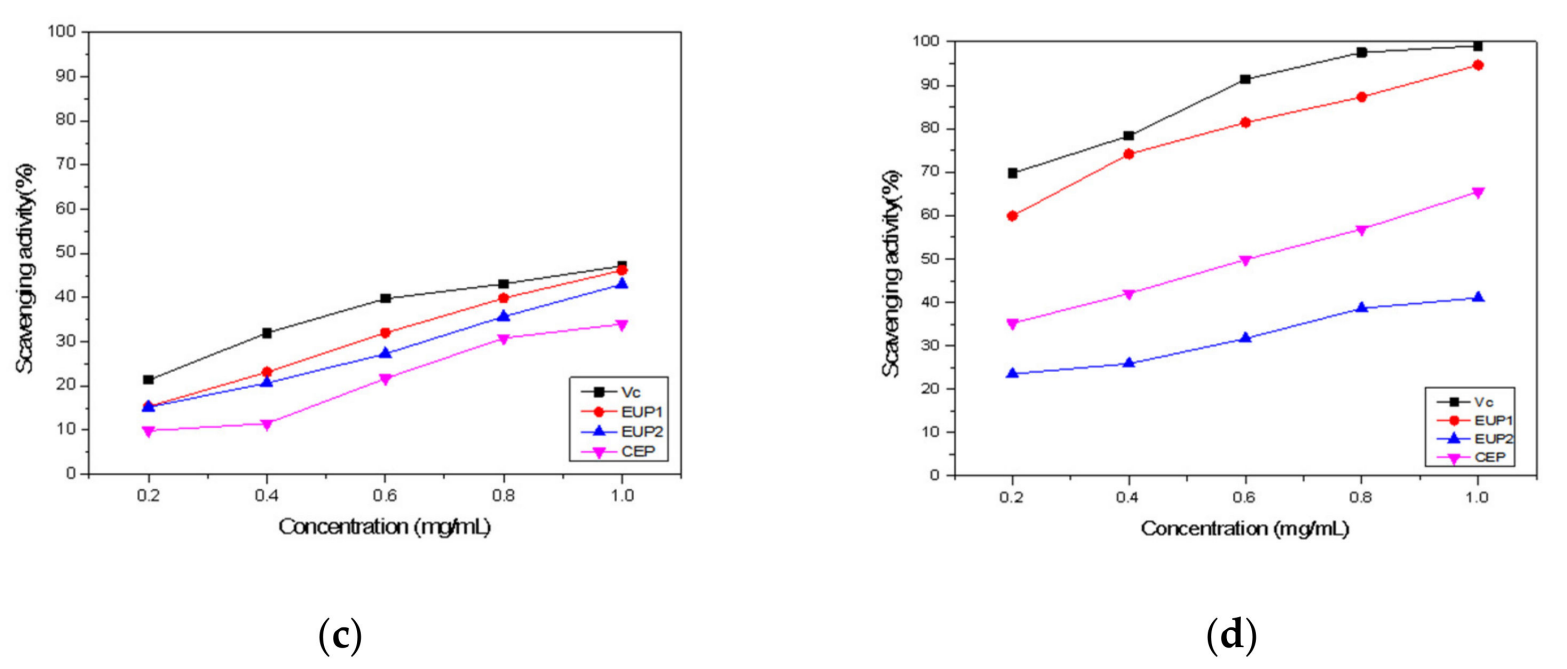
Antioxidant activity of various polysaccharide fractions extracted from *Elaeagnus umbellata*. (**a**) DPPH• radical scavenging assay; (**b**) •OH radical scavenging assay; (**c**) ABTS•+ assay; and (**d**) O^2−^ assay.

The DPPH• radical has been widely used to evaluate the free radical scavenging capacity of natural compounds by donating hydrogen to form a stable DPPH• molecule [25]. This radical has a characteristic absorbance at 517 nm and exhibits a purple color that tends to fade when the radical is scavenged by the antioxidant [26]. When DPPH• encounters a proton-donating substance, the radical is scavenged and the absorbance at 517 nm decreases [27]. Figure 5a shows that the scavenging activities of EUP1, EUP2, and CEP increased as the concentration of the sample increased from 0.2 to 1.0 mg/mL. The scavenging effects of EUP1, EUP2, and CEP were 91.31%, 69.49%, and 65.34%, respectively, at 1.0 mg/mL. The antioxidant activities of EUP1 were satisfactory, with a dose–effect relationship found among the concentrations and DPPH• scavenging effects. Figure 5a shows that EUP1 had a more significant effect on scavenging DPPH• radicals than EUP2 and CEP.

#### 2.2.2. Hydroxyl Radical Scavenging Ability

•OH radicals are considered the most harmful free radicals among reactive oxygen species and can damage various macromolecules in the human body, such as carbohydrates, nucleic acids, lipids, and amino acids [28]. Therefore, removing hydroxyl radicals is an important method of antioxidant defense. The scavenging effects of EUP1, EUP2, and CEP on hydroxyl radicals (Figure 5b) at 1.0 mg/mL were 95.01%, 71.59%, and 46.21%, respectively. Both EUP1 and EUP2 exhibited inhibitory effects on •OH radicals. The scavenging ability of the purified polysaccharide was significantly greater than that of the crude polysaccharide. The •OH scavenging activity for polysaccharides from a lichen, Umbilicaria esculenta, has been reported as 67.1% at 4 mg/mL [29]. Regarding •OH removal, the polysaccharides may act as electron or hydrogen donors to scavenge the hydroxyl radicals, with previous studies reporting that removing the generated •OH is the basis of the antioxidant mechanism [30].

#### 2.2.3. ABTS•+ Radical Scavenging Activity 

The ABTS•+ radical scavenging activity assay is considered simple and quick in operation and has been used to measure the total antioxidant capacity of polysaccharides [31]. The ABTS•+ radical scavenging activity assay resulting from the present study (Figure 5c) showed similarly high values of 46.22%, 43.01%, and 34.02% at 1 mg/mL for EUP1, EUP2, and CEP, respectively—values similar to that for Vitamin C.

#### 2.2.4. Superoxide Anion Scavenging Activity 

The superoxide anion, a type of precursor to singlet oxygen, is a resilient radical produced by various biological and photochemical reactions [32]. Superoxide anion radicals can react with many types of biomolecules to produce more reactive oxidative species, which can damage DNA and cause many diseases [33]. The superoxide anion radical scavenging activities of EUP1, EUP2, and CEP were less than those of Vitamin C but increased at a higher concentration, reaching 94.65%, 41.02%, and 65.49%, respectively, at a concentration of 1.0 mg/mL (Figure 5d). Overall, EUP1 exhibited the strongest ability to scavenge superoxide anion radicals.

## 3. Discussion

Due to the wide range of sources and certain functions of natural polysaccharides, they can serve as bioactive substances to regulate the physiological functions of the body, and they have minimal toxic and side effects. Natural polysaccharides with complex structures extracted from edible resources are widely used in clinical medicine and have attracted the attention of researchers both domestically and internationally. *E. umbellata* is well known for its high content of lycopene in its berries, with antioxidant and anti-inflammatory activity. Since the content of polysaccharides in its berries is relatively lower than of lycopene, the physiological activity of its polysaccharides has not been explored. Polysaccharides, which are essential biological macromolecules, have been widely used as food stabilizers, thickeners, emulsifiers and texture modifiers. Recently, research interest in polysaccharides has increased because of their various physiological properties, such as their antioxidant, immunomodulatory, antitumor, and tranquilizing activities. In this study, a crude polysaccharide with high yield was extracted from *E. umbellata*, and the extracted crude polysaccharide was separated. The two components were further purified by a Sephadex G-100 gel column to obtain EUP1 and EUP2. The molecular weights of EUP1 and EUP2 were determined by high-performance gel permeation chromatography (HPGPC), and the antioxidant activities of EUP1, EUP2 and CEP in vitro were studied.

Polysaccharides are polar substances; polar solvents are often used to extract polysaccharides. Generally speaking, extracted plant polysaccharides belong to crude polysaccharides, and low molecular-weight compounds and inorganic salts are removed by methods such as water dissolution and ethanol washing. Then, proteins are removed through the Sevag method, an enzyme or freeze-thaw process, etc., then decolorized, and finally, column chromatography such as gel filtration chromatography, ion exchange chromatography or affinity chromatography is used to further purify and finally obtain purified polysaccharides [34]. The further separation and purification of deproteinized crude polysaccharides was performed on a DEAE-52 column using anion exchange chromatography. The total recovery rate of crude polysaccharides applied to this column was 81.51%. Then, Sephadex G-100 was used to further purify the final EUP1 and EUP2, with yields of 43.68% and 20.85%. Niyomploy et al. separated crude polysaccharides from wild turmeric by DEAE cellulose ion exchange column chromatography and purified it by Superdex G-200 gel filtration column chromatography to obtain two relatively rich polysaccharides, P_11_ and P_21_ [35].

There are certain differences in the biological activity of polysaccharides with different molecular weights, and the molecular weight range of polysaccharides from different sources is different. Shen Yu et al. [36] separated five different molecular-weight polysaccharides from the fruit of Acanthopanax senticosus through ultrafiltration and found that polysaccharides with different molecular weights had varying degrees of antioxidant activity by measuring their clearance rates for DPPH and superoxide radicals. Among them, the polysaccharides with molecular weights ranging from 50 to 10 kDa had the highest antioxidant activity, with clearance rates of 56.89% and 40.69%, respectively. Low molecular-weight polysaccharides have more significant antioxidant activity, possibly because at the same mass, lower molecular-weight polysaccharides have more reducing hydroxyl terminals and are able to accept and scavenge more free radicals [37]. In addition, the type and molar ratio of monosaccharides in polysaccharides can have a certain impact on their antioxidant activity [38]. For example, arabinose and galactic acid contribute significantly to the scavenging activity of polysaccharides against DPPH free radicals, while polysaccharides with high levels of fucose, rhamnose, and uronic acid have stronger scavenging ability against hydroxyl free radicals [39]. In this study, the molecular weights of EUP1 and EUP2 were determined to be 63 kDa and 38 kDa, respectively, by high-performance gel permeation chromatography (HPGPC). High-performance liquid chromatography (HPLC) showed that the monosaccharide composition and molar ratio of EUP1 and EUP2 were different. The in vitro antioxidant activity of EUP1, EUP2, and CEP was studied, and the results showed that EUP1 had higher antioxidant activity and the purified milk polysaccharides were superior to crude polysaccharides in scavenging free radicals. EUP1 has high scavenging activity against DPPH radicals, hydroxyl radicals, ABTS radicals and superoxide anions. In these aspects, EUP2 is not as strong as EUP1, but it also has moderate clearance capabilities. Despite the favorable in vitro potential of any compound or extract, the safety level and the beneficial outcomes could only be determined through in vivo toxicological studies. 

## 4. Materials and Methods

### 4.1. Materials and Chemicals

*Elaeagnus umbellata* fruits were obtained from the PingLu County Forestry Bureau (Shanxi Province, China). Selected ripe *Elaeagnus umbellata* fruits were washed with water, and then, the seeds were removed. After drying at 60 °C for 6 h, the fruits were crushed into a powder and then sieved through a 40–65 mesh screen. The final moisture content of the dried fruit pulp powder was 13.76%. The 95% ethanol, phenol and concentrated sulfuric acid were of analytical grade, provided by Tianjin Fengchuan Chemical Reagent Technology Co., Ltd. (Tianjin, China). DEAE-52 and Sephadex G-100 were purchased from Sigma Chemicals Co. (St. Louis, MO, USA). Sodium chloride, 1-butanol and chloroform were all analytical pure. A series of standard monosaccharides came from J&K Chemicals Technology Co., Ltd. (Beijing, China); 1-phenyl-3-methyl-5-pyrazolone (PMP) and Tri-fluoroacetic acid (TFA) came from Tokyo Chemical Industry Co., Ltd. Dimethyl sulfoxide (DMSO) came from Shanghai Yi’en Chemical Technology Co., Ltd. (Shanghai, China), Mica sheet (Ȼ 9.9 mm) came from Xiangyuan electronic materials Co. Ltd. (Jiangsu Province, China). DPPH (2,2-diphenyl-1-picrylhydrazyl) was purchased from Sigma (St. Louis, MO, USA), and all other reagents used were of analytical grade ABTS (2,2′-azinobis-(3-ethylbenzothiazoline-6-sulphonic-acid) diammomium-salt).

### 4.2. Instruments and Equipment

Electronic analytical balance was from Mettler Toledo Technology Co., Ltd. (Shanghai, China). TDL-5C centrifuge was from Anting Scientific Instruments Co., Ltd. (Shanghai, China). SP-2000UV Ultraviolet visible spectrophotometer was from Shanghai Spectral Instrument Co., Ltd. (Shanghai, China).

### 4.3. Extraction Procedure

A sample of the processed raw materials (3.00 g) was weighed and then placed in an Erlenmeyer flask. After enzymolysis, the enzymes were inactivated, and homogenate extraction was conducted using hot water [40,41]. After centrifugation (4500× *g*, 15 min), the supernatant was concentrated, added to 4 times the volume of absolute ethanol, and then placed in a refrigerator at 4 °C for alcohol precipitation (12 h). After centrifugation (4500× *g*, 15 min), the precipitate was dried to yield the crude polysaccharide (CEP).

### 4.4. Purification of the Extracted Polysaccharide

#### 4.4.1. DEAE-Cellulose 52

The proteins were removed from the CEP using the Sevag method [42]. The crude polysaccharide solution (10 g/L) was passed through a DEAE-cellulose 52 column (20 mm × 400 mm). The polysaccharides were fractionated and eluted using a linear gradient of 0, 0.1, 0.2, 0.3 and 0.4 M NaCl-Tris-HCl buffer solutions at a flow rate of 1.0 mL/min. The eluted polysaccharide solution was collected in consecutive 3 mL samples from each tube. Using this method, two polysaccharide fractions were obtained and labeled as EUP1 and EUP2. The total sugar concentration in each tube was determined by the phenol-sulfuric acid procedure by monitoring the absorbance at 490 nm [43]. The purified polysaccharide fractions were finally lyophilized for the structural and antioxidant activity analyses.

#### 4.4.2. Sephadex G-100 Gel Chromatography

The two fractions exhibiting the highest and symmetrical peak profiles were collected and lyophilized to obtain the purified polysaccharides for further purification by gel filtration chromatography. The polysaccharide sample was loaded onto a Sephadex G-100 gel column (16 mm × 300 mm) at a flow rate of 1.0 mL/min. A 0.1-M NaCl buffer solution was used as the eluent with 3 mL collected in each tube at 0.5 mL/min. The fractions were collected and then combined in accordance with the results of analysis by the phenol–sulfuric acid procedure by monitoring the absorbance at 490 nm. The main peak was recorded then dialysis and lyophilization were used to obtain the pure polysaccharide.

### 4.5. Characterization of Polysaccharides

#### 4.5.1. UV and FT-IR Spectroscopy

Ultraviolet visible spectroscopy studies the valence electron structure and chemical bond strength of substances by measuring their absorption of ultraviolet light. The Fourier transform infrared spectrometer utilizes the characteristic of the sample absorbing infrared light. The position and intensity of infrared absorption peaks reflect the characteristics of molecular structure, which can be used to identify the structural composition of unknown substances or to determine their chemical groups. The absorption intensity of the absorption band is related to the content of chemical groups and can be used for quantitative analysis and purity identification. The polysaccharide fractions were prepared as 0.5 mg/mL aqueous solutions. A UV-Vis spectrophotometer was used to record the UV spectra of the samples at wavelengths ranging between 200 and 400 nm. For the FT-IR analysis, about 1 mg of the dried polysaccharides was mixed with about 100 mg of dried KBr powder and then pressed into a pellet. The FT-IR spectra of the polysaccharides were obtained over a frequency range from 4000 to 500 cm^−1^ on a Thermo Scientific Nicolet iS50 FTIR spectrometer (Thermo Electron Scientific Instruments LLC, Madison, WI, USA).

#### 4.5.2. Determination of Molecular Weight

The molecular weights (MW) of EUP1 and EUP2 were determined by high-performance gel permeation chromatography (HPGPC). The HPGPC system was equipped with a TSK gel GMPWXL column (Tosoh Corporation, Yamaguchi Prefecture, Japan, 7.8 mm × 300 mm, 13 µm) and an RI-201H differential refractometer (Shodex China Co. Ltd., Shanghai, China) as the detector. The conditions were as follows: injection volume 20 μL, column temperature 35 °C, using distilled water (H_2_O) at a flow rate of 1.0 mL/min. Dextrans of different molecular weights (T-180, T-2000, T-4600, T-7100, T-10000, T-21400, T-84400, T-133800, and T-2000000) were used as standards, from which a standard curve based on the following equation was established:

(1) $$\mathrm{logMW}\;=\;-0.867t_{R}\;+\;13.302\left(R^{2}\;=\;0.9925\right)$$

where t_R_ is retention time. 

#### 4.5.3. Analysis of Monosaccharide Composition

As described in recent studies, the polysaccharides were hydrolyzed by TFA (trifluoroacetic acid); then, the obtained monosaccharides were derivatize by PMP (1-phenyl-3-methyl-5-pyrazolone) [44,45]. The analysis was performed using a 1260 Infinity II HPLC system (Agilent Technologies, Santa Clara, CA, USA) equipped with an InfinityLab Poroshell 120 EC-C [46] column (Agilent Technologies, 150 mm × 4.6 mm) and UV detection at a wavelength of 250 nm. A mixture of acetonitrile (A) and 0.06 mol/mL phosphate bluffer solution (B) was used as the mobile phase at a ratio of 18:82 (*v*/*v*). A 20 μL volume of the sample solution was injected and then separated on the column at 40 °C at a flow rate of 1.0 mL/min. Each monosaccharide was identified by comparison with the corresponding standards.

#### 4.5.4. Scanning Electron Microscopy (SEM)

The morphological features of EUP1 and EUP2 were observed using a field-emission scanning electron microscope (SU8100, Hitachi, Tokyo, Japan). A small sample of polysaccharides was dispersed on a round aluminum sheet, sputtered with a 20 nm thick gold film under vacuum, observed by SEM with an acceleration voltage of 5 KV, and photographed at different magnifications.

#### 4.5.5. Thermal Gravimetric Analysis (TGA)

Analysis was performed using a thermal gravimetric analyzer (STA 409, NETZSCH-Gerätebau GmbH, Selb, Germany) as described previously [47], Two 5 mg freeze-dried samples were weighed on the TGA microbalance and then heated at 30 °C/min from 30 to 700 °C. Nitrogen gas was used as the heating medium at a flow rate of 100 mL/min.

### 4.6. Analysis of Antioxidant Activities

#### 4.6.1. DPPH• Radical Scavenging Activity

Scavenging the DPPH free radical is a common method for measuring antioxidant capacity. The DPPH• radical scavenging activity of the polysaccharide was measured as described previously [48,49]. In brief, 2 mL of DPPH• anhydrous ethanol solution (0.2 mM) was added to 2 mL of the polysaccharide sample solutions (0.2, 0.4, 0.6, 0.8, and 1.0 mg/mL); then, 2 mL of water was added to the tube. The mixture was then shaken and kept in the dark at room temperature for 30 min. The absorbance of the supernatant was measured at 517 nm on a UV-visible spectrophotometer, with Vitamin C used as a positive control. The DPPH• radical scavenging activity was calculated using the following equation:

(2) $$\mathrm{DPPH}\cdot \;\mathrm{scavenging}\;\mathrm{effect}\left(\%\right)=\frac{\left[1-\left(A_{2}-A_{1}\right)\right]}{A_{0}}\times 100$$

where A_0_ is the absorbance of the DPPH• solution without the sample; A_2_ is the absorbance of the sample mixed with the DPPH• solution; and A_1_ is the absorbance of the sample without the DPPH• solution, i.e., the control.

#### 4.6.2. •OH Scavenging Ability 

The •OH scavenging activity was measured as described previously [50], with some modifications. In brief, 1.0 mL of the polysaccharide sample solutions (0.1, 0.4, 0.8, 1.2, 1.6, and 2.0 mg/mL) was blended with 1.0 mL of FeSO_4_ (2 mmol/L) and 1.0 mL of salicylic acid-ethanol solution (6 mmol/L); then, 1.0 mL of H_2_O_2_ (6 mmol/L) was added to the mixed solution before incubation at 37 °C in a water bath for 30 min. The absorbance of the mixture was immediately measured at 510 nm using an UV-vis spectrophotometer. Vitamin C was used as the positive control. The hydroxyl radical scavenging activity was calculated using the following equation:

(3) $$\mathrm{Hydroxyl}\;\mathrm{scavenging}\;\mathrm{activity}\left(\%\right)=\frac{\left[1-\left(A_{2}-A_{1}\right)\right]}{A_{0}}\times 100$$

where A_0_ is the absorbance of the reagent blank; A_1_ is the absorbance of the control; and A_2_ is the absorbance of the sample.

#### 4.6.3. ABTS•+ Radical Cation Scavenging Activity 

The ABTS•+ radical cation scavenging activity was determined as described previously [51]. The working solution was prepared by mixing two stock solutions (25 mL ABTS stock solution (7 mmol/L) and 440 μL potassium persulfate (140 mmol/L)) together and then allowing them to react for 12–16 h at room temperature in the dark. At the time of determination, the ABTS•+ working solution was prepared by diluting the stock solution with anhydrous ethanol until its absorbance at 734 nm was 0.7 ± 0.02. Polysaccharide sample solutions (10 μL) over a concentration range from 0.2 to 1.0 mg/mL were mixed with the ABTS•+ working solution and left at room temperature for 6 min. The absorbance was then measured at 734 nm, with Vitamin C as the reference compound. The ABTS•+ radical scavenging activity of the sample was calculated as follows:

(4) $$\mathrm{ABTS}•+\;\mathrm{radical}\;\mathrm{scavenging}\;\mathrm{activity}\left(\%\right)=\frac{1-\left(A_{2}-A_{1}\right)}{A_{0}}\times 100$$

where A_0_ is the absorbance of the blank reagent; A_1_ is the absorbance of the control (ABTS•+ solution without test sample); and A_2_ is the absorbance in the presence of the test sample. 

#### 4.6.4. Superoxide Anion Scavenging Activity 

The modified Marklund method [52] was used to determine the superoxide anion scavenging ability of EUP. Briefly, 4.5 mL of Tris-HCl buffer solution (50 mol/L, pH 8.2) and 1.0 mL of the sample solution were incubated at 25 °C in a water bath for 10 min. Pyrogallol (0.1 mL, 25 mol/L) incubated at 25 °C was added quickly; then, the absorbance of the mixture was measured at 320 nm, using Vitamin C as a positive control. The superoxide anion scavenging activity was calculated using the following formula:

(5) $$\mathrm{Superoxide}\;\mathrm{anion}\;\mathrm{scavenging}\;\mathrm{activity}\left(\%\right)=(1-\frac{A_{1}}{A_{0}})\times 100$$

where A_0_ is the absorbance of water alone, and A_1_ is the absorbance of the sample.

## 5. Conclusions

Curd polysaccharides from *Elaeagnus umbellata* were purified through DEAE-cellulose 52 and Sephadex G-100 gel chromatography, consisting of two fractions: EUP1 and EUP2. The average MW of the EUP1 and EUP2 polysaccharides were 63 and 38 kDa, respectively. EUP1 mainly consisted of L-rhamnose and D-galactose at a molar ratio of 2.05:1, and EUP2 consisted of D-mannose, L-rhamnose, D-galactose, and D-arabinose at a molar ratio of 2.06:1:2.78:1. The FT-IR analysis of EUP1 and EUP2 showed that they were acidic polysaccharides. EUP also possessed significant scavenging activities against the DPPH, ABTS, superoxide anion and hydroxyl radicals. Therefore, EUP could be developed as a potential antioxidant agent for application in the fields of functional foods or medicine. These results may also provide a theoretical basis for the wider use of EUP in antioxidative products and in the pharmaceutical industries.

## Data Availability

All data generated or analyzed during this study are included in this manuscript.

## Sample Availability

Samples of the compounds are not available from the authors.
